# Exploring temperature-dependent transcriptomic adaptations in *Yersinia pestis* using direct cDNA sequencing by Oxford Nanopore Technologies

**DOI:** 10.1038/s41598-025-05662-1

**Published:** 2025-07-01

**Authors:** Brandon Robin, Alexandre Baillez, Servane Le Guillouzer, Cécile Lecoeur, Florent Sebbane, Sébastien Bontemps-Gallo

**Affiliations:** Univ. Lille, CNRS, Inserm, CHU Lille, Institut Pasteur de Lille, U1019 - UMR 9017 - CIIL - Center for Infection and Immunity of Lille, 59000 Lille, France

**Keywords:** Oxford Nanopore Technology, RNA-Seq, *Yersinia pestis*, Operons mapping, Temperature adaptation, Gene expression, Bacteria

## Abstract

Transcriptomics is key to understanding how bacterial pathogens adapt and cause disease, but remains constrained by cost, technical, and biosafety issues, especially for highly virulent and/or regulated pathogens. Here, we present a streamlined and cost-effective RNA-Seq workflow using Oxford Nanopore Technologies for direct cDNA sequencing, suitable for complete in-house implementation. This method avoids PCR bias, enables multiplexing, and includes built-in quality controls and alignment benchmarking. Applied to *Yersinia pestis* (the causative agent of plague), the workflow produced an experimentally validated operon map and revealed novel transcriptional units, including within the pathogenicity island. Transcriptomic profiling at 21 °C and 37 °C, modeling the flea and mammalian environments, highlighted temperature-driven metabolic shifts, notably the upregulation of sulfur metabolism and the *dmsABCD* operon. These findings provide insights into *Y. pestis* adaptation and illustrate how long-read RNA-Seq can support operon discovery, genome annotation, and gene regulation studies in high-risk or understudied bacterial pathogens.

**Supplementary Information:**

The online version contains supplementary material available at 10.1038/s41598-025-05662-1.

## Introduction

Understanding bacterial pathogenesis is crucial to anticipating and managing emerging and re-emerging infectious diseases. In this context, transcriptomic analysis has proven invaluable for exploring how pathogens adapt to diverse environments, evade immune defenses, and acquire antibiotic resistance^1–3^. However, traditional approaches, such as short-read RNA sequencing, often require costly and specialized equipment and are typically confined to centralized research platforms^4^. These technical and logistical constraints are even more pronounced when working with highly pathogenic bacteria, where strict biosafety regulations govern the handling and transfer of live organisms and their genetic material^5^. Together, these combined technical and biosafety constraints highlight the need for alternative methodologies that are both cost-effective and fully implementable within secured laboratory settings.

Among the pathogens for which such alternative methodologies are needed is *Yersinia pestis*, the causative agent of plague. This highly virulent bacterium alternates between two markedly different environments, the flea vector and mammalian hosts, requiring rapid and precise transcriptional reprogramming to ensure survival^1,3,6–8^. Temperature is one of the key environmental cues that trigger these transcriptomic profile shifts^9,10^. It is therefore not surprising that better knowledge of these shifts is essential to understanding the molecular mechanisms of *Y. pestis* adaptation to its host and vector.

To address technical and biosafety constraints while enabling high-resolution transcriptomic analysis of highly regulated pathogens such as *Y. pestis*, and to shed light on the molecular mechanisms this bacillus uses to establish infection, we developed a detailed workflow based on Oxford Nanopore Technologies (ONT) for direct cDNA sequencing. This protocol integrates the latest ONT R10 chemistry with multiplexing (SQK-LSK114 and SQK-NBD114.24) and includes benchmarking of two widely used alignment tools (Bowtie and Minimap2) to ensure accurate transcript alignment. We notably applied this workflow to *Y. pestis*, a highly regulated pathogen for which the method was specifically developed. Cultures were grown at 21 °C and 37 °C, temperatures representative of the flea and mammalian environments, respectively, allowing us to generate an experimentally validated operon map and capture gene expression changes associated with host adaptation.

## Results and discussions

### Direct cDNA PCR-free sequencing workflow

ONT-based RNA-Seq can be performed using the Direct RNA, PCR-cDNA, or Direct cDNA approaches^11^. The Direct RNA approach is the most appealing for preserving native RNA characteristics. However, the lack of multiplexing solutions makes it particularly expensive^12^. This limitation can be mitigated by using a PCR-based approach, although it introduces bias, leading to the over-representation of certain sequences and the under-representation of others^13,14^. To balance performance, cost, and bias reduction, we opted for a Direct cDNA sequencing tailored to enterobacterial models^11^. The protocol spans from total RNA extraction to sequencing and remains compatible with other bacterial species following appropriate optimization of the bacterial cell lysis step. Each step of the workflow was designed with cost-effectiveness in mind, guiding the selection of commercial kits to ensure both reliability and affordability.

**Fig. 1:**
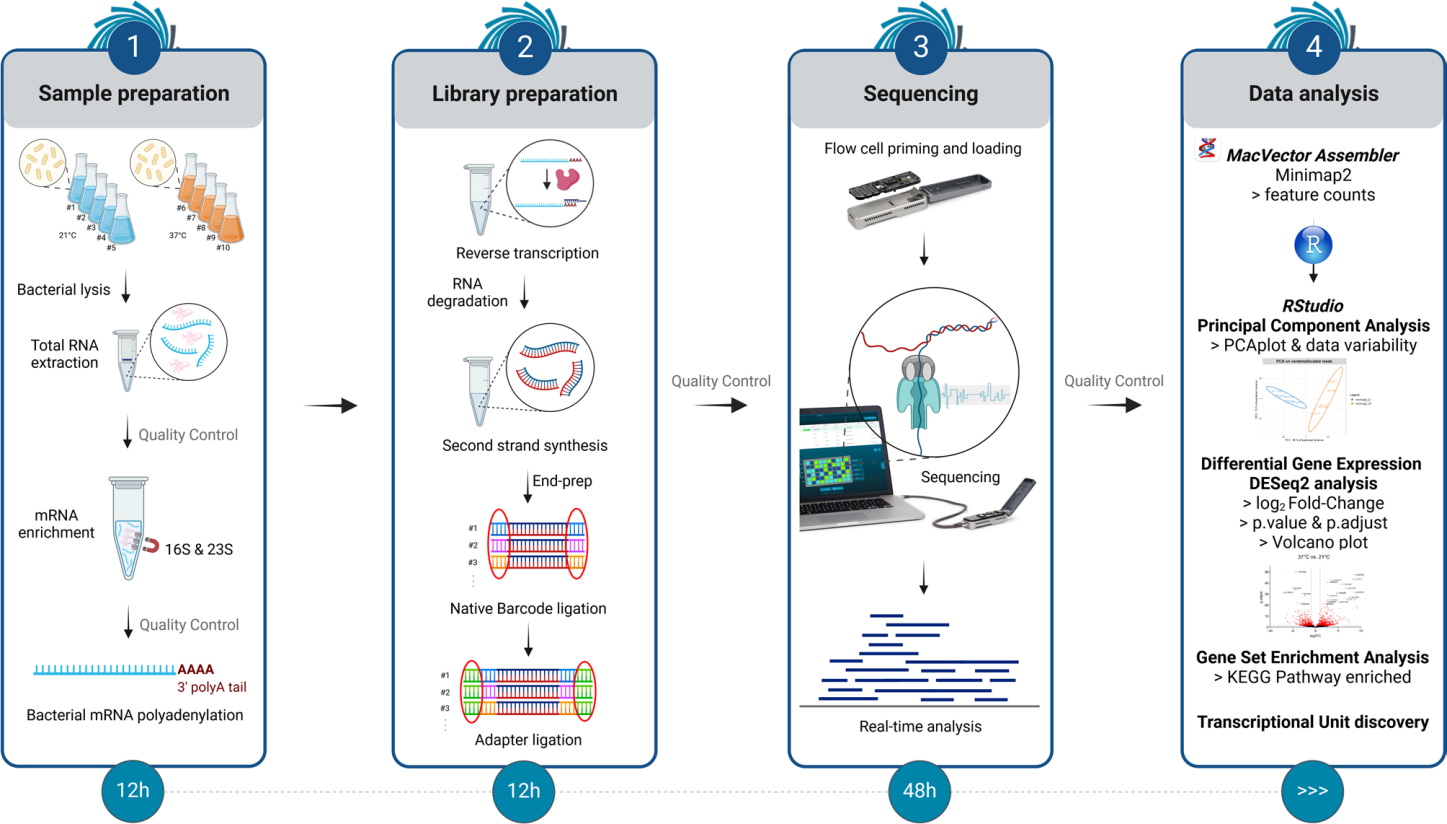
Direct cDNA sequencing workflow. The workflow comprises four main steps that include sample preparation, library preparation, sequencing, and computational data analysis. Sample preparation includes bacterial growth, cell lysis, total RNA extraction, ribosomal RNA depletion (16S and 23S), and in vitro polyadenylation to ensure compatibility with the Oxford Nanopore cDNA protocol. Library preparation involves reverse transcription with strand switching, RNA degradation, second-strand synthesis, end-repair, native barcoding, and adapter ligation. Sequencing is performed on a MinION device after flow cell priming and setup in MinKNOW, enabling real-time data acquisition. Computational analysis comprises basecalling, alignment to a reference genome, quantification of gene expression, principal component analysis (PCA), differential expression analysis (DESeq2), gene set enrichment analysis (GSEA), and operon identification. Figure created with BioRender (https://BioRender.com/k62c145).

The workflow (Fig. 1) is divided into four parts: sample preparation, library preparation, sequencing, and data analysis, requiring approximately 12, 12, and 48 h, respectively, with data analysis time varying depending on the methods used. The detailed step-by-step protocol is available on Protocols.io.

*Sample preparation*. Five independent cultures were grown at 21 °C and 37 °C and immediately mixed with RNAprotect Bacteria Reagent (Qiagen) to stabilize and preserve RNA. After centrifugation, the cell pellets were treated with lysozyme to improve lysis efficiency, followed by the addition of the lysis buffer from the Nucleospin RNA extraction kit. RNA quantity, purity (260/280 ratio), and integrity of the purified RNA were assessed using a Qubit fluorometer (offering better performance than a Nanodrop spectrophotometer), a Nanodrop spectrophotometer, and a Bioanalyzer, respectively (Fig. S1a). Quality controls were performed at each stage, and samples failing to meet quality criteria were excluded from further processing. To improve sequencing depth, ribosomal RNA (16S and 23S), which constitutes approximately 90% of total RNA, was depleted using the MICROBExpress kit (Ambion). Although this kit demonstrates slightly lower depletion efficiency compared to other commercial options^15^, it was chosen for its cost-effectiveness. It successfully removed around 95% of the 16S and 23S rRNA (Fig. S1b), resulting in a fourfold enrichment of mRNA and a 150% increase in mRNA detection sensitivity^16^. As the ONT Direct cDNA protocol requires RNA with a polyadenylated 3′ end, and a minor fraction of bacterial RNAs are polyadenylated^17^, the enriched RNA samples were polyadenylated in vitro using *Escherichia coli* poly(A) polymerase, as recommended by ONT^18^. The polyadenylated RNA was then purified using SparQ PureMag beads (Quantabio), a cost-effective alternative to the more commonly used but expensive AMPure XP beads (Beckman Coulter).

*Library preparation*. For the reverse transcription and second-strand synthesis steps of library construction, we used the ONT SQK-LSK114 protocol with slight modifications. Notably, instead of the LongAmp Taq DNA polymerase (designed for synthesizing fragments up to 30 kb) originally recommended, we used the Q5 High-Fidelity DNA polymerase (NEB), which offers greater fidelity and is suitable for synthesizing DNA fragments up to 20 kb. This choice provided both sufficient processivity and improved sequence accuracy in our PCR-free workflow, given that the median size of bacterial transcripts is around 900-bp, with most operons ranging from 500-bp to 3-kb. For the second part of the library preparation (i.e., from end-preparation to flow cell loading), we used the ONT SQK-NBD114.24 protocol. We combined both protocols to enable native barcoding and allow the simultaneous sequencing of up to 24 samples, significantly reducing costs, an advantage not currently available with the Direct RNA sequencing protocol. Importantly, this approach also avoided PCR amplification, thereby minimizing bias in transcript representation and preserving the relative abundance of enriched bacterial mRNAs. For the end-preparation step, which includes phosphorylation of the 5′ ends and the addition of a 3′ dA-tail, protocol adjustments were necessary because the input material consisted of cDNA obtained from second-strand synthesis rather than PCR amplicons initially expected in the SQK-NBD114.24 protocol. In particular, the quantity of DNA Control Sample has been proportionally adapted to the lower input quantity of cDNA provided by the SQK-LSK114 protocol. Each sample was then individually barcoded using a unique tag (barcodes 1 to 24) and ligated to ONT sequencing adapters. These adapters are pre-loaded with a motor protein and a hydrophobic tether, which together facilitate the controlled translocation of the cDNA through the nanopores during sequencing. Lastly, the final library was purified using Short Fragment Buffer rather than Long Fragment Buffer, as it allows retention of DNA fragments across the full size range. In other words, this choice avoids bias toward long fragments (> 3 kb) and is better suited to the typical size distribution of bacterial transcripts.

*Sequencing*. The prepared library (100 fmol) was loaded onto a primed ONT flow cell (R10 chemistry), using the recommended buffer with the optional addition of bovine serum albumin (BSA) to enhance sequencing performance. One of the key advantages of ONT technology is real-time data acquisition and alignment monitoring via the MinKNOW software. To enable this, the reference genome FASTA file was preloaded into MinKNOW prior to sequencing. Before launching the sequencing run, the setup was configured using MinKNOW’s interface, which allows several optional customizations, including barcode demultiplexing, quality score filtering, and read length thresholds. In our case, we selected the fast basecalling mode to ensure real-time performance and reduce the risk of computational overload during extended runs. Once initiated, the run can be stopped at any point based on the desired depth of coverage. Typically, a minimum of 10 × is required to achieve approximately 75% gene coverage in *E. coli* K12^10^. In our case, sequencing was extended to at least 48 h to maximize read depth and yield the most informative dataset. As a result, the flow cell could not be reused, unlike in shorter runs where early stopping may preserve its integrity.

### Post-sequencing quality controls and data analyses

**Fig. 2:**
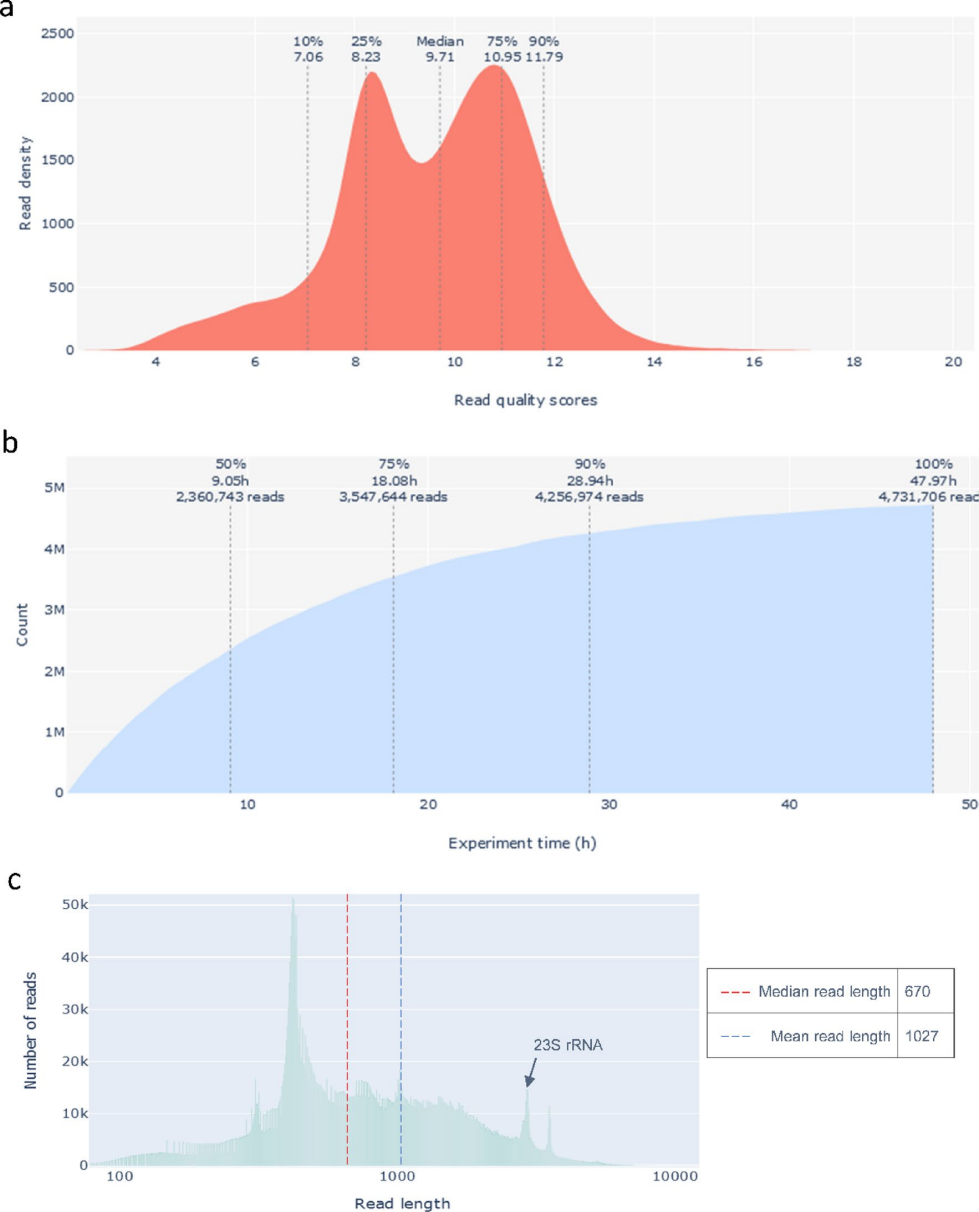
Visualization of post-sequencing quality metrics using PycoQC and NanoPlot. (**a**) Read quality score distribution and (**b**) cumulative data yield were produced with PycoQC. (**c**) Read length distribution was generated using NanoPlot.

To improve read accuracy, we re-basecalled the data after the run using either the high-accuracy or super-accuracy mode. In addition to the real-time data displayed by MinKNOW, the sequencing summary file was also uploaded to Galaxy (https://usegalaxy.org/) for further exploration. Post-run metrics were visualized using PycoQC^19^ and Nanoplot^20^ (Fig. 2), which enabled a detailed and intuitive overview of sequencing performance. These tools allowed us to assess key quality indicators, including the cumulative yield over time (Fig. 2a), the distribution of read quality scores (Fig. 2b), and the read length distribution (Fig. 2c), each providing useful insights into the run’s dynamics and data structure. Together, these visualizations helped us confirm that the run proceeded smoothly and yielded data consistent with the expected transcript length and quality.

In our dataset, approximately 50% of the reads were generated within the first 10 h of sequencing, and 90% were acquired by 30 h (Fig. 2a). The median read quality score was 10, with a range from 0 to 18 (Fig. 2b). Median and mean read lengths were 670 and 1027 nucleotides, respectively (Fig. 2c), consistent with expected bacterial mRNA sizes. For all downstream analyses, we retained only reads with a quality score greater than 8, corresponding to an estimated basecalling accuracy of ~ 90%.

Reads retained after quality filtering (Q > 8) were automatically sorted into GNU zipped archive compressed FASTQ files, each labeled with the corresponding sample barcode. Adapter trimming was performed during basecalling, ensuring that the reads were ready for alignment without additional preprocessing. The alignment was performed using two widely available tools, namely Bowtie, an ultrafast memory-efficient short-read aligner^21^, and Minimap2, a more recent aligner optimized for long, noisy RNA-Seq reads^22^. We initially tested Bowtie because our read length (~ 670 nt) falls within its upper performance range, and subsequently compared its results to those obtained with Minimap2, which is better suited for long-read RNA-Seq data. While Bowtie failed to align approximately 6% of the retained reads, resulting in an 18% data loss and a coverage loss greater than 10x, Minimap2 successfully aligned the full dataset. Despite this, the impact on gene identification was modest: both aligners recovered about 90% of genes, with Minimap2 detecting, on average, 0.7% more genes (Table 1).

**Table 1:** Comparison of read alignment and gene identification in *Y. pestis* samples grown at 21 °C and 37 °C using Bowtie and Minimap2.

		WT1_21	WT2_21	WT3_21	WT4_21	WT5_21	WT1_37	WT2_37	WT3_37	WT4_37	WT5_37
Bowtie	Aligned reads	209946	185954	168175	208557	176633	163065	186568	157146	174408	155964
	Unaligned read	17150	30037	5665	17562	10180	9268	6151	6844	4854	7567
	Total reads	227096	215991	173840	226119	186813	172333	192719	163990	179262	163531
	Avg coverage depth	33	30	29	33	32	30	32	31	32	28
	genes	3809	3776	3647	3674	3705	3739	3766	3712	3716	3724
Minimap2	Aligned reads	720974	666000	663530	830686	624028	515559	593075	531074	608036	526906
	Unaligned read	0	0	0	0	0	0	0	0	0	0
	Total reads	720974	666000	663530	830686	624028	515559	593075	531074	608036	526906
	Avg coverage depth	44	41	40	43	45	41	45	43	46	38
	genes	3812	3794	3702	3711	3742	3753	3786	3766	3756	3735
Differences	Coverage depth	11	11	11	10	13	11	13	12	14	10
	genes	3	18	55	37	37	14	20	54	40	11
	%	0.07	0.42	1.30	0.87	0.87	0.33	0.47	1.27	0.94	0.26

**Fig. 3:**
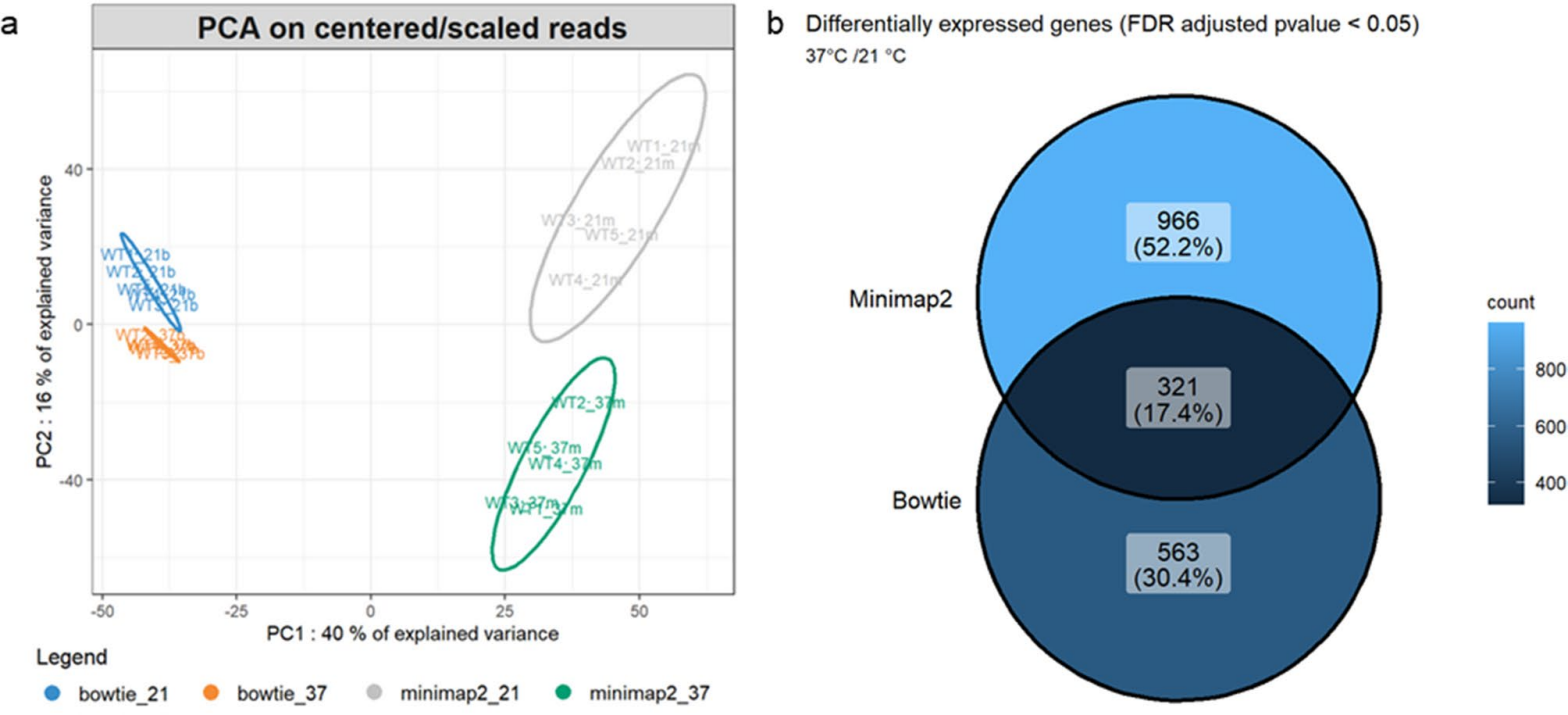
Impact of alignment tool (Bowtie vs Minimap2) on transcriptomic data variability and differential gene expression. (**a**) PCA of transcriptomic data processed with Bowtie and Minimap2. The ellipses represent the 95% confidence intervals around each group. Samples grown at 21 °C are shown in blue (Bowtie) and grey (Minimap2), while samples grown at 37 °C appear in orange (Bowtie) and green (Minimap2). (**b**) Venn diagram showing the overlap of differentially expressed genes identified by DESeq2, depending on the alignment tool used.

To assess whether the choice of alignment tool could influence biological interpretations, we compared results obtained with Bowtie and Minimap2 using principal component analysis (Fig. 3a). The two main components clearly separated the samples according to both alignment method (Bowtie vs. Minimap2) and growth temperature (21 °C vs. 37 °C). Replicates were tightly clustered within each condition, although slightly more variability was observed in the Minimap2 group. This variability was also reflected in the differential expression analysis (Fig. 3b). Using the same input data, Bowtie and Minimap2 identified 563 and 966 differentially expressed genes, respectively. Among them, 321 genes (17%) were shared between the two aligners. The lower number of genes detected with Bowtie likely results from its limited ability to align longer reads, particularly those above 1000 nucleotides (Fig. 2c) and are better handled by Minimap2. In total, 645 genes were uniquely identified by Minimap2, supporting its superior sensitivity and making it more suitable for transcriptomic analyses based on long-read sequencing technologies such as Oxford Nanopore. Altogether, these results highlight the importance of selecting an aligner compatible with the characteristics of long-read RNA-Seq data, and support the use of Minimap2 for accurate transcript quantification in ONT-based workflows.

### The experiment-based operon landscape

**Fig. 4:**
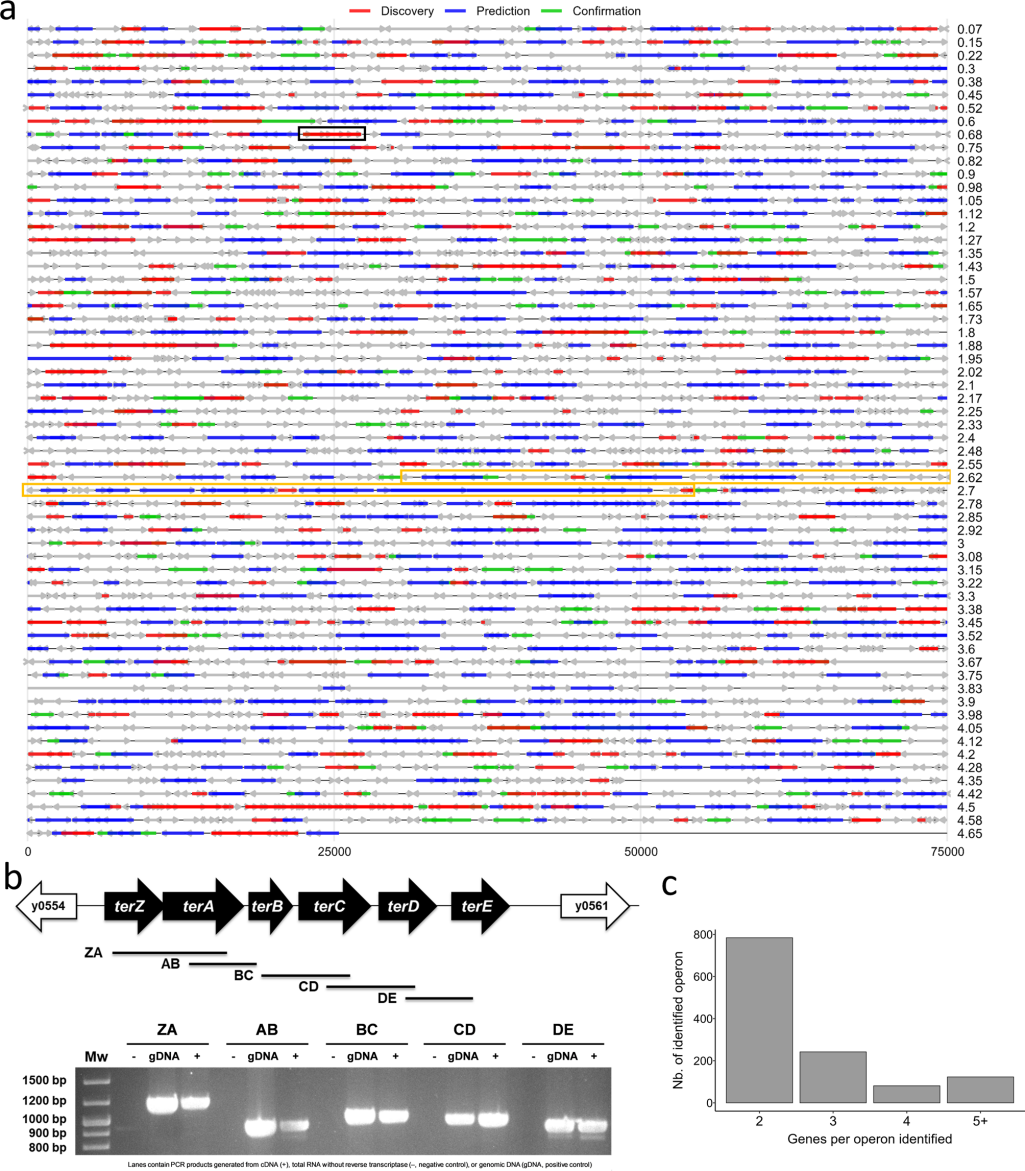
Genome-wide operon map of *Y. pestis*. (**a**) The circular chromosome of *Y. pestis* KIM10 + (4.6 Mb) was divided into 62 consecutive 75-kb segments to facilitate visualization of gene positions and operon organization. Grey arrows indicate individual genes not forming operons. Red, blue, and green arrows represent operons identified only in our dataset (“Discovery”), only predicted by MicrobesOnline (“Prediction”), or shared by both sources (“Confirmation”), respectively. The black and yellow boxes highlight the validated *terABCDE* operon and the *pgm* locus, respectively. (**b**) Experimental validation of the *terABCDE* operon by RT-PCR. The gene organization is illustrated schematically, with arrows showing gene orientation and black bars indicating the amplified regions (labeled ZA, AB, BC, CD, DE). These labels correspond to the bands observed on the adjacent agarose gel. Lanes contain PCR products generated from cDNA ( +), total RNA without reverse transcriptase (–, negative control), or genomic DNA (gDNA, positive control). Original gel is presented in Supplementary Fig. 2 (**c**) Histogram illustrating how operons are distributed according to their number of constituent genes, from two to five or more.

Although long-read sequencing with ONT theoretically allows each read to correspond to a single transcriptional unit (i.e., one read = one transcriptional unit), it remains limited in its ability to accurately define bacterial operons. This is due to the loss of strand specificity, the lack of 5′-end resolution (preventing identification of transcriptional start sites), and biases introduced during reverse transcription. Despite these constraints, we successfully generated (as described in the material and method section) a transcriptional map using data from the ten sequenced samples (Fig. 4a). Supporting the robustness of our approach for identifying polycistronic transcriptional units, we confirmed by RT-PCR that all six genes of the well-characterized *terZABCDE* operon were co-expressed^23^ (highlighted in black in Fig. 4a; see also Fig. 4b). In addition to *terZABCDE*, other well-characterized operons such as *phoPQ* and *ureABC*, also showed transcriptional patterns consistent with co-expression, further supporting the reliability of the method.

Building on this validation, we performed a genome-wide analysis of our sequencing data to determine the number, structure, and gene composition across the *Y. pestis* genome. We identified 1230 operons, most of which consisted of two genes (784 operons), while the largest extended to as many as 16 genes (Fig. 4c). Of these, 763 could be compared to the 1365 operons listed in the MicrobesOnline database for the *Y. pestis* KIM chromosome, which are based solely on adjacent gene pairs (i.e. it only detects operon composed of two genes). The remaining 446 operons were not comparable, as they included more than two genes and thus exceeded the pairwise format used in the database. Among the 763 comparable operons, 463 matched predicted structures (“Confirmation”, shown in green in Fig. 4a), while 321 were newly discovered in our dataset (“Discovery”, red). In contrast, 902 operons predicted by MicrobesOnline were not detected in our analysis (“Prediction”, blue in Fig. 4a). Taken together, our data identified 767 operons that were not predicted by MicrobesOnline, either because they were entirely novel or because they exceeded the prediction model’s constraints. These findings reveal both the limitations of in silico prediction models and the enhanced resolution offered by long-read transcriptomic analysis.

To further illustrate the biological value of our operon-level analysis, we focused on the 102-kb unstable *pgm* locus of *Y. pestis*, which comprises the pigmentation segment and a high-pathogenicity island (HPI), both required for successful infection of the flea vector and the mammalian host. Among the 28 operons predicted by MicrobesOnline (i.e. operons of two genes) within the *pgm* locus, only two of the 28 operons (namely, *astCA* and *hmsRS*) were also identified in our data, while three others (*phoH*/Y_RS12135, Y_RS12325/Y_RS12330, and Y_RS12410/Y_RS22565/Y_RS12420) were newly discovered using our experimental approach (orange frame, Fig. 4a; Table S1). Lastly, in addition to the operons discussed above, 1637 genes were not included in any predicted or experimentally identified operon (grey in Fig. 4a), suggesting that a substantial portion of the genome may be transcribed as monocistronic units or under yet-undefined conditions. Overall, our results demonstrate how long-read transcriptomics can refine operon architecture beyond conventional prediction models, offering a more accurate and comprehensive view of gene organization in bacterial genomes.

### Temperature-driven transcriptomic adaptation of *Y. pestis*

*Y. pestis* cycles between a mammalian host (primarily rodents) and its flea vector. As such, transmission between host and flea, and the associated temperature shifts from 37 °C to ambient temperature and back, acts as a key environmental cue sensed by the bacterium to regulate gene expression and adapt to each host niche^3,7,9,10^. We therefore used our ONT-based RNA-Seq workflow to compare the global gene expression profiles of *Y. pestis* KIM6 + cultured at 37 °C and 21 °C, the latter being optimal for the development of a transmissible infection in fleas^6^.

**Fig. 5:**
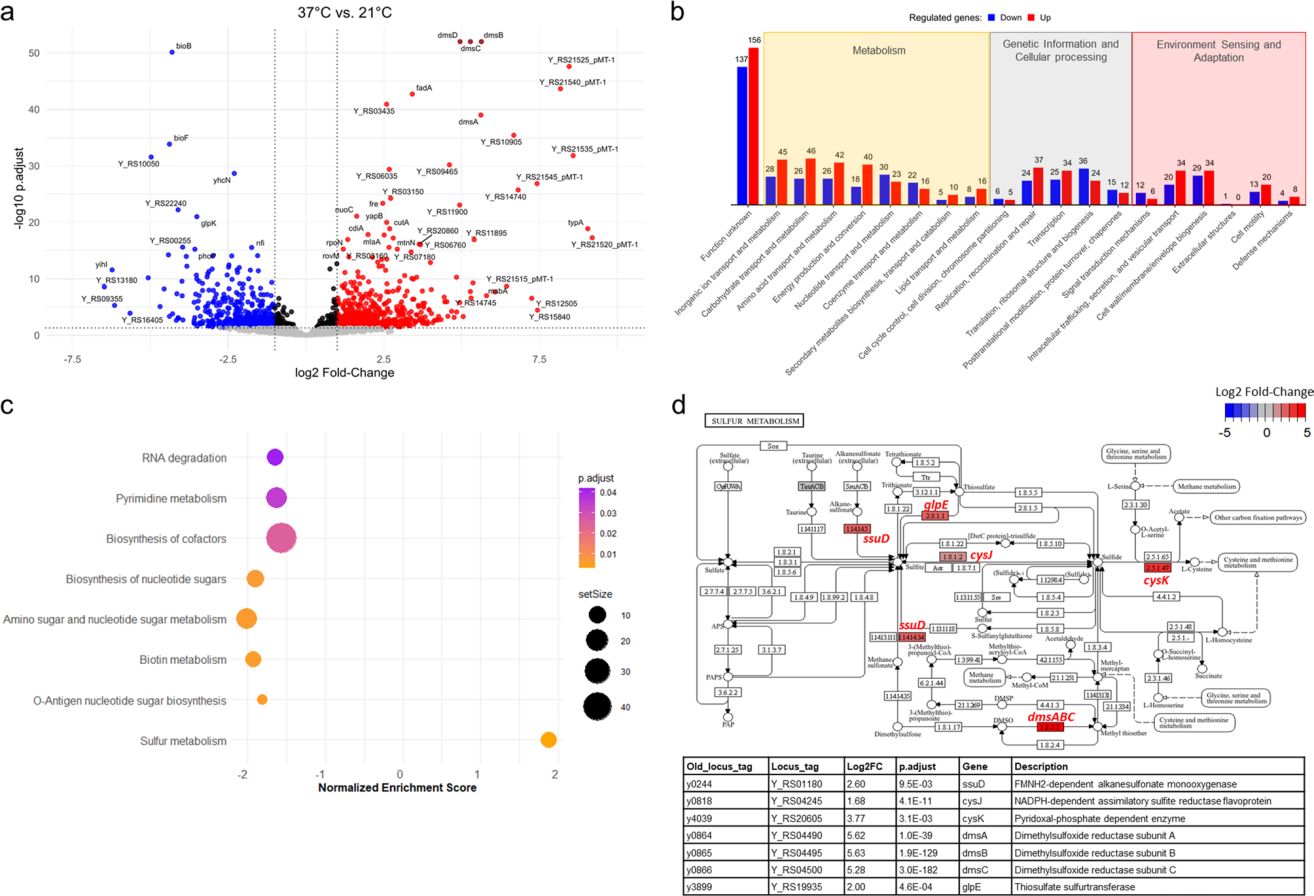
Temperature-dependent transcriptomic response of *Y. pestis* highlights sulfur metabolism induction at 37 °C. (**a**) Volcano plot illustrating gene regulation between 21 and 37 °C. Grey or black, red, and blue dots represent genes with no significant change (adjusted p-value ≥ 0.05 or − 1 < log₂ fold-change < 1), significantly upregulated (log₂ FC > 1), and downregulated (log_2_ FC < –1) genes, respectively. Labeled dots correspond to the 50 most regulated genes based on p-value and fold-change. (**b**) Functional classification of significantly regulated genes according to COG categories. Bar colors indicate upregulated (red) and downregulated (blue) genes, and numbers above bars indicate gene counts per category. (**c**) Gene Set Enrichment Analysis (GSEA) of differentially expressed genes using KEGG pathways. The dot plot shows pathways ranked by normalized enrichment score (NES). Dot size represents the number of genes per pathway, and color intensity reflects adjusted p-values. Most pathways were negatively enriched (left side), with the exception of sulfur metabolism, which showed positive enrichment at 37 °C. (**d**) KEGG sulfur metabolism pathway overlaid with gene expression data (The figure was generated based on data from the KEGG database^46^). Genes significantly upregulated at 37 °C are labeled in red. The corresponding table lists locus tags, fold-changes, adjusted p-values, and functional annotations of the most impacted genes in this pathway.

We identified 1287 genes differentially expressed between 37 and 21 °C (Fig. 5a), with 14.4% of annotated genes upregulated and 12.0% downregulated at 37 °C. These proportions are consistent with previous analyses conducted in *Y. pestis* strains EV76 and 195/P, which exhibited 1387 and 1038 differentially expressed genes, respectively, under similar conditions^7,10^. This consistency suggests that the overall scale of the temperature-dependent transcriptional response may be conserved across strains and biovars. Indeed, KIM6 + belongs to the Medievalis biovar, whereas EV76 and 195/P are part of the Orientalis biovar^24,25^. As these biovars represent distinct evolutionary lineages, functional comparisons will be required to disentangle conserved from lineage-specific regulatory programs.

To further investigate the biological functions impacted by temperature, we first performed a functional classification of differentially expressed genes based on Clusters of Orthologous Genes (COG) categories (Fig. 5b). This analysis revealed substantial metabolic reprogramming at 37 °C. To refine these findings, we performed Gene Set Enrichment Analysis (GSEA) using KEGG pathways (Fig. 5c). Most significantly enriched pathways had negative normalized enrichment scores (NES), indicating that the corresponding genes were predominantly downregulated at 37 °C. These included pathways related to metabolism and envelope biosynthesis, such as amino sugar and nucleotide sugar metabolism, pyrimidine metabolism, biotin metabolism, and RNA degradation. Notably, the *so-called* pathways for O-antigen nucleotide sugar biosynthesis (NB: *Y. pestis* is missing a O-side chains on its LPS) and cofactor biosynthesis were also strongly downregulated at 37 °C. The latter involved 47 genes, accounting for 34% of the pathway. These findings point to a temperature-dependent remodeling of surface antigens consistent with previous reports describing structural modifications at 37 °C^26–30^.

Among the 50 most strongly impacted genes, 37 were upregulated and 13 downregulated at 37 °C compared to 21 °C (Fig. 5a; Table S2, highlighted in grey). Notably, the four genes of the *dmsABCD* operon ranked among the ten most highly induced loci at 37 °C. This operon which governs the reduction of DMSO to dimethyl sulfide, thereby supporting anaerobic respiration through the use of sulfur-based electron acceptors^31^. Interestingly, our GSEA analysis also revealed the upregulation of several genes associated with sulfur metabolism, including *ssuD*, *cysJ*, *cysK*, and *glpE*, although these were not among the top-ranked genes (Fig. 5d). These genes encode enzymes involved in distinct aspects of sulfur assimilation and stress resistance^32–36^. SsuD facilitates the use of alkanesulfonates as sulfur sources under sulfate limitation; CysJ and CysK participate in cysteine biosynthesis, a key sulfur-containing amino acid; and GlpE catalyzes sulfur transfer reactions that may mitigate oxidative stress^32–36^. Taken together, these observations suggest that sulfur metabolism is upregulated at 37 °C as part of a broader adaptive strategy supporting redox homeostasis, anaerobic respiration, and bacterial persistence under host-like conditions. This interpretation is further supported by previous studies showing that *dmsABC* is overexpressed in infected rat lymph nodes (buboes)^3,7^, and that a *dmsABC* deletion mutant is outcompeted in pooled mutant screens in a rat model of bubonic plague^8^.

## Conclusion

We present a complete RNA-Seq workflow using Oxford Nanopore Technologies for transcriptomic analysis in bacterial systems requiring biosafety-compliant handling. This streamlined approach integrates PCR-free direct cDNA sequencing, multiplexing, and quality control steps, and was benchmarked using two alignment tools. While relying on existing ONT chemistries, the workflow provides a pragmatic and reproducible solution for studying gene expression in pathogens under containment constraints. Applied to *Y. pestis*, the method enabled the identification of both conserved and novel operons, including within the pathogenicity island, underscoring the value of long-read sequencing for refining genome annotations. Transcriptomic profiling across flea- and mammal-relevant temperatures further revealed temperature-driven reprogramming of core metabolic pathways, notably the upregulation of sulfur metabolism and the *dmsABCD* operon. Overall, this approach offers a practical alternative for transcriptomic studies of high-risk or undercharacterized bacteria, particularly when standard short-read workflows are not feasible due to cost, infrastructure, or biosafety limitations.

## Methods

### Bacterial strain and growth conditions

The avirulent strain *Y. pestis* KIM6 + , which is a derivative of the KIM10 + strain, was used^37^. It lacks the pYV plasmid. This bacillus was cultured in Lysogeny Broth (LB) at either 21 °C or 37 °C with shaking. Cultures were inoculated at OD_600_ = 0.05 and grown until reaching OD_600_ ≈ 0.7 ± 0.05. The 37 °C culture reached OD_600_ = 0.7 after 7 h of incubation, while the 21 °C culture reached the same OD after approximately 8 h.

### RNA-Seq workflow

The detailed step-by-step protocol is available on protocols.io (10.17504/protocols.io.8epv5rkw5g1b/v1). Briefly, total RNA was extracted from five biological replicates per temperature condition using the NucleoSpin RNA kit (Macherey–Nagel), followed by mRNA enrichment through depletion of 16S and 23S rRNA using the MICROBExpress kit (Thermo Fisher). Enriched RNA was polyadenylated using *E. coli* poly(A) polymerase (NEB), then purified with SparQ PureMag beads (Quantabio). Library preparation was performed using ONT protocols SQK-LSK114 and SQK-NBD114.24, with modifications. This included reverse transcription, second-strand synthesis using the Q5 High-Fidelity DNA Polymerase (NEB), end-preparation, native barcode multiplexing, and adapter ligation. Sequencing was carried out on a MinION Mk1B device using a FLO-MIN114 R10.4.1 flow cell and controlled with MinKNOW software v. 23.11.2. Sequencing data have been deposited in the NCBI Sequence Read Archive under accession number PRJNA1220679.

### Principal components and differential gene expression analyses

PCA and differential expression analyses were performed in R (v4.4.1) using RStudio (2023.12.1 Build 402). Transcript counts per gene and condition were obtained using Bowtie or Minimap2 algorithms available in MacVector software (v18.7). PCA was carried out with the pca function from the mixOmics package, to assess sample clustering by experimental condition^38^. Differential gene expression between the two temperature conditions (37 °C vs. 21 °C) was assessed using the DESeq2 package^39^. Raw counts were normalized using the DESeq2 median-of-ratios method, which calculates size factors to account for differences in library depth across samples. Genes with zero counts across all samples in either condition were removed (802 genes for Bowtie, 707 for Minimap2) and genes with adjusted p < 0.05 were considered significantly differentially expressed.

### Operon identification and comparative mapping

Operon analysis was conducted in R (v4.4.1) using RStudio (2024.04.2 Build 764), with GenomicAlignments^40^, GenomicRanges^40^, and Tidyverse^41^ packages. Reads were imported from BAM files using readGAlignments and genomic features from the *Y. pestis* KIM10 + GFF file (NCBI) via import using the rtracklayer package^42^. Overlapping reads from the 10 pooled samples and features were identified using findOverlaps with a ≥ 100 bp threshold. Reads were converted into genomic ranges with the GRanges function. PTUs were defined as reads spanning at least two consecutive genes in the correct order and retained only if supported by ≥ 100 overlapping reads (average ≥ 10 reads/sample) with queryHits and diff functions from base R. Duplicate PTUs, defined by identical start and end coordinates, were filtered using duplicated from base R. The listed operons were compared to 1365 gene-pair-based predictions from the MicrobesOnline database (http://www.microbesonline.org/). Common and unique operons were identified using intersect and setdiff. Visualization was done using ggplot2.

### RT-PCR for *ter* operon validation

Total RNA was extracted using TRIzol Reagent (Invitrogen), followed by chloroform separation, isopropanol precipitation, and ethanol washing. RNA was resuspended in RNase-free water and treated with TURBO DNase (Ambion) at 37 °C for 30 min. Reverse transcription was performed on 20 ng RNA using SuperScript III (Invitrogen) with random hexamers and RNase inhibitors. The resulting cDNA was treated with RNase H at 37 °C for 20 min. PCR (35 cycles) was carried out with DreamTaq DNA Polymerase (Thermo Scientific) on 4 μL of cDNA, total RNA (i.e. control without reverse transcriptase), or genomic DNA (positive control of amplification) in a final volume of 20 μL. PCR products (10 μL) were mixed with loading buffer and resolved on 2% agarose gels.

### COG functional category attribution, gene set enrichment analysis and KEGG pathway visualization

COG categories were assigned using eggNOG-mapper (v2.1.12)^43^. Genes significantly regulated (adjusted p < 0.05) were ranked by log₂ fold-change and analyzed using GSEA via the gseKEGG function (clusterProfiler)^44^, with gene set size limits (min = 3, max = 300) and p-value cutoff = 0.05. Results were visualized as dot plots with ggplot2^47^, and KEGG pathway maps were generated using pathview^45^, overlaying gene expression values (e.g., sulfur metabolism) onto colored KEGG diagrams.

## Electronic supplementary material

Below is the link to the electronic supplementary material.

Supplementary Material 1

Supplementary Material 2

Supplementary Material 3

Supplementary Material 4

Supplementary Material 5

## Data Availability

Sequencing data have been deposited in the NCBI Sequence Read Archive under accession number PRJNA1220679.
