# Supplementary material for: Exploring temperature-dependent transcriptomic adaptations in Yersinia pestis using direct cDNA sequencing by Oxford Nanopore Technologies
Source: Sci Rep. 2025 Jul 1;15:20564. doi: 10.1038/s41598-025-05662-1 (PMC12218796; doi:10.1038/s41598-025-05662-1)
Supplement: Supplementary file 4 — Supplementary Material 4 [file 41598_2025_5662_MOESM4_ESM.docx]

**Supplementary information**

**Figure S1. Representative electropherograms generated by the Agilent 2100 Bioanalyzer showing nucleic acid quality and library size.** (a) Evaluation of total RNA integrity after extraction. (b) Assessment of 16S and 23S rRNA depletion efficiency prior to polyadenylation. (c) Estimation of cDNA library size distribution before sequencing.

**Figure S2. Original gel images corresponding to the data shown in Figure 4b.**

**Table S1. Identified operons in *Y. pestis* based on long-read RNA-Seq data and comparison with MicrobesOnline predictions.** This table lists operons identified in our dataset, with genomic coordinates (min_start, max_end), segmental positions (start_fragment, end_fragment), segmental coordinates (start_position, end_position), old locus tags, gene names, and functional descriptions. The final column indicates whether the operon is confirmed, discovered, or unmatched (-) with MicrobesOnline predictions. The yelllow frame highlights the *pgm* locus, which contains the high-pathogenicity island. min_start and max_end refer to chromosomal coordinates. start_fragment and end_fragment indicate the 75-kb genomic segments used in Fig. 4. start_position and end_position define the operon’s position within these segments.

**Table S2. Differentially expressed genes in *Y. pestis* at 37 °C compared to 21 °C.** This table lists all genes found to be significantly differentially expressed (adjusted p-value < 0.05). The top 10 genes, ranked by p-value and absolute log₂ fold-change, are highlighted in light grey, and the top 50 are shaded in dark grey. Font color indicates the direction and magnitude of expression change; red (log₂ fold-change > 1), dark red (> 2), blue (< –1), and dark blue (< –2). Genes with modest fold changes (–1 to 1) are shown in light grey text.
