# Supplementary figures and images for: Exploring temperature-dependent transcriptomic adaptations in Yersinia pestis using direct cDNA sequencing by Oxford Nanopore Technologies
Source: Sci Rep. 2025 Jul 1;15:20564. doi: 10.1038/s41598-025-05662-1 (PMC12218796; doi:10.1038/s41598-025-05662-1)

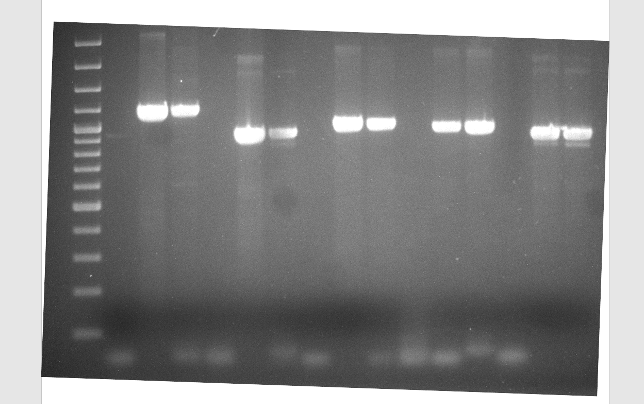

Supplement: Supplementary file 5 — Supplementary Material 5 [file 41598_2025_5662_MOESM5_ESM.png]
